# A Transmission Power Optimization with a Minimum Node Degree for Energy-Efficient Wireless Sensor Networks with Full-Reachability

**DOI:** 10.3390/s130303951

**Published:** 2013-03-20

**Authors:** Yi-Ting Chen, Mong-Fong Horng, Chih-Cheng Lo, Shu-Chuan Chu, Jeng-Shyang Pan, Bin-Yih Liao

**Affiliations:** 1 Department of Electronic Engineering, National Kaohsiung University of Applied Sciences, Kaohsiung 807, Taiwan; E-Mails: ytchen@bit.kuas.edu.tw (Y.-T.C.); jspan@cc.kuas.edu.tw (J.-S.P.); byliao@cc.kuas.edu.tw (B.-Y.L.); 2 Chung-Shan Institute of Science and Technology, Zuoying 90008, Taiwan; E-Mail: louccc@hotmail.com; 3 School of Computer Science, Engineering and Mathematics, Flinders University, Adelaide 5068, Australia; E-Mail: scchu@bit.kuas.edu.tw

**Keywords:** transmission optimization, minimum node degree, energy-efficient, full reachability networks

## Abstract

Transmission power optimization is the most significant factor in prolonging the lifetime and maintaining the connection quality of wireless sensor networks. Un-optimized transmission power of nodes either interferes with or fails to link neighboring nodes. The optimization of transmission power depends on the expected node degree and node distribution. In this study, an optimization approach to an energy-efficient and full reachability wireless sensor network is proposed. In the proposed approach, an adjustment model of the transmission range with a minimum node degree is proposed that focuses on topology control and optimization of the transmission range according to node degree and node density. The model adjusts the tradeoff between energy efficiency and full reachability to obtain an ideal transmission range. In addition, connectivity and reachability are used as performance indices to evaluate the connection quality of a network. The two indices are compared to demonstrate the practicability of framework through simulation results. Furthermore, the relationship between the indices under the conditions of various node degrees is analyzed to generalize the characteristics of node densities. The research results on the reliability and feasibility of the proposed approach will benefit the future real deployments.

## 1. Introduction

### 1.1. The Development of the Wireless Sensor Network

Since the 1990s, wireless sensor networks (WSNs) have attracted attention from academics and industries because of their applications in environmental monitoring, healthcare, automation and the Internet of Things (IoT). According to ONWorld, the world market for wireless sensor network (WSN) systems and services will increase to approximately 46 billion US dollars, which is 500 million US dollars more than the present level [1]. In wireless sensor networks, a large number of nodes are typically deployed in a region. Each node is capable of sensing and delivering measurement data. The deployed nodes are self-configured and communicate by radio to form a network topology. The nodes directly or indirectly transmit data to data sinks. In a wide-deployed WSN, direct delivery is not always suitable because battery-powered nodes cannot establish a direct link to data sinks. As a result, indirect delivery is typical in WSNs. In such an environment, an energy-efficient network topology with full reachability is desirable. However, there are certain restrictions on the design and operation of nodes in WSNs. For example, each node must be tiny, low cost, long-life, robust and reachable in a topology that can operate in a harsh environment. If equipped with these features, WSNs will be more broadly applicable. WSNs are a fundamental network as a part of IoT. Thus, wireless sensor networks are regarded as significant to the future IoT to be developed.

### 1.2. Research Motivation and Goal

In wireless sensor networks, topology control is an important research area. Ideal topology control benefits reachability and energy efficiency. The primary objectives of topology control in wireless sensor networks are to maintain network reachability, rather than optimizing network lifetime. In the past, network connectivity was considered as an evaluation of network quality and reachability are used as performance indices to evaluate the connection quality of a network. The connectivity is defined as the percentage of direct connections between arbitrary node pairs in a network. Through direct links, nodes communicate with each other in a hop. However, full connectivity does not guarantee the quality of network service. For an example, power consumption and signal interference become serious when each node will transmit packets in a high power level to reach all nodes. Instead, a hop-by-hop approach is more efficient than that of full connectivity. The reachability is the percentage of either indirect or direct delivery between any nodes. Each node is able to communicate with all distant nodes through relay nodes. In other works, there is no any isolated node in the full reachability network. And the necessary transmission range of each node in a full connectivity network is greater than it in a full reachability network. Network reachability is directly affected by connectivity. Network connectivity is affected by node degree, which is the number of connected neighboring nodes. In an open space, node degree is controlled by the transmission power of nodes. The node degree increases when the transmission range is increased by a higher transmission power. However, the energy-saving performance is reduced. Network reachability is poor when the connectivity of the network is too low. High connectivity will consume more power and decrease the quality of service. How to determine the optimal transmission power of a node is a critical question investigated in [2,3].

The goals of this research on optimal transmission power control of nodes include the realization of full reachable network and the improvement of energy efficiency. In this study, a transmission optimization with a minimum node-degree algorithm is proposed to construct an energy-efficient wireless sensor network with full reachability. The objective of constructing a fully reachable network topology is to guarantee the transmission routes of any node pair and avoid creating unreachable nodes. A wireless sensor network with a minimum node degree can be constructed according to the proposed algorithm. Additionally, an optimal wireless sensor network will be energy-efficient. Furthermore, the proposed algorithm adjusts the transmission range according to various node deployment densities. This study analyzes the relationships between the transmission power and reachability with different densities. Finally, simulations are performed to verify the energy efficiency and full reachability of the proposed approach.

### 1.3. Organization

The rest of this paper is organized as follows: in Section 2, a survey of the previous developments of wireless sensor networks is presented. In Section 3, an algorithm for transmission optimization with a minimum node degree is proposed to attain full reachability in a wireless sensor network topology. In Section 4, to verify the performance of the proposed algorithm, a series of simulations is designed and conducted to examine the impact factors including node density, node degree and node distribution on energy efficiency and reachability. In Section 5, the relationship of energy efficiency and reachability with different node densities is discussed. Finally, in Section 6, we summarize and suggest perspectives for future work.

## 2. Related Work

### 2.1. Application of Wireless Sensor Networks

A wireless sensor network is composed of a large number of nodes that can sense wireless communication. The nodes in a wireless sensor network collect environmental information from the region of deployment by sensor and deliver data wirelessly to data sinks. Environmental monitoring, forest-fire prevention, and healthcare are examples of traditional WSN applications. In these applications, the wireless sensor networks only monitor, detect and communicate. However, in the last decade, wireless sensor network applications have increasingly been the object of study, and innovative applications have been developed. One promising new application is the Internet of Things (IoT). According to the International Telecommunication Union (ITU), the IoT will enable collaboration and communication between people and things and between things themselves, which was previously unimagined [4]. The system enable the connection of objects equipped with sensors over the Internet, which can subsequently sense one another using an identification protocol and communicate. Most objects on the IoT are embedded with RFID, a sensor or a wireless communication chip. Compared with a WSN, the IoT is “smarter” because it can process, analyze and respond to the environmental context, rather than only sense and deliver contextualized real-world information.

IoT architecture is composed of three layers: sense, network and application. The sense layer is composed of accessible wireless devices with any type of sensor, such as a RFID sensor and wireless sensor nodes. In a wireless sensor network, topology construction is the first step. The nodes deliver the information through neighboring nodes in the network to form an arbitrary network topology. However, an arbitrary network topology is not suitable for all WSN applications. Therefore, a flexible network topology is valuable for a wireless sensor network. A network topology is constructed by topology control according to network information to adjust transmission power with the goal of enhancing service performance and prolonging the lifetime of a network. Topology control is a critical technique in wireless sensor networks and IoT. In sum, an energy-efficient network topology with full reachability is a fundamental requirement in prolonging the lifetime and maintaining the quality of service for WSN and IoT.

### 2.2. A Critical Question in the Development of Wireless Sensor Networks

A wireless sensor network is a type of distribution system and is composed of a large number of specially designed sensors [5–7]. Battery power is the main power supply for sensors in a WSN. The power consumption and workload of each sensor are not balanced. Additionally, the transmission range and work power of each node are strictly limited. The task of a wireless sensor network is control and monitoring in the deployment area under adverse circumstances for a long period of time. The node is rarely maintained, replaced or charged because of deployment conditions. The performance of the network will be affected when the power of node is exhausted. Therefore, the question of how to prolong network lifetime and maintain network Quality-of-Service (QoS) is important. In fact, QoS is a technique of network resource management to provide the stable and predictable transmission quality for user requirements. QoS of network as a guarantee of service quality in a network is easily influenced by link quality. An oversupply of links causes significant channel interference, whereas fewer links lead to fail in discovering the links to compose a reachable route to the destination. Thus, how to find a certain set of links is a significant step to establish a QoS-enabled WSN. Recently, the researches on QoS [8,9] had been proposed to construct an optimal WSN through topology control [10] and a routing algorithm [11,12]. The traditional evaluation indices of QoS are defined by latency, jitter, loss and throughput of packets in transmission. However, as the evolution of wireless sensor networks, the reachability and energy efficiency of nodes have become ones of important issues in QoS [13–18]. The problem of network lifetime arises because a WSN will not operate properly if the network has too many disabled sensors. This problem implies that the lifetime of a network is related to the energy efficiency of the nodes in networks. In this paper, we will explore the relationship between node reachability and network topology. That will be critical to the QoS of WSNs.

### 2.3. WSN Architecture

According to function and logical organization, there are three types of network architecture: flat [19], hierarchical [20,21] and hybrid [22]. A flat network is known as a non-hierarchical network, of which the property and function are the same for all sensors, such as RIS [23], MSNL [24], LADS [25] and ASCEMT [26]. A hierarchical network is a network in which all sensor nodes are clustered through the cluster technique according to property and function [27,28]. Every cluster is composed of at least one sensor, and each sensor in a cluster has a corresponding cluster head, which collects data from the sensors in the cluster and transmits the data to a data sink. Hierarchical networks facilitate equalized power consumption. In addition, the sensors in a cluster are located close to one another. The data collected from sensors in the same cluster are similar. Data aggregation will reduce data transmissions, thereby reducing power consumption and prolonging network lifetime. Concepts based on the hierarchical network to save and reduce power and equalize power consumption by sensors include LEACH [29], LDS [30] and HEED [31].

In hierarchical networks, Low Energy Adaptive Clustering (LEACH) is the most well-known distribution cluster algorithm. In this approach, the cluster heads alternate, and periodicity is selected through threshold adjustment to equalize power consumption. Initially, each sensor has two probability and threshold values between zero and one. The sensor will be a cluster head if the probability is less than a threshold. The threshold will be zero if the sensor is a cluster head in the present period. This cluster head node will not be selected as a cluster head in the next period. A threshold of sensor will be raised to increase the possibility of being selected as a cluster head. When a node has never been a cluster head, the node will be forced to become a cluster head by setting its threshold to zero. LEACH produces a large number of broadcast packages and repetitive information, which can cause broadcast storms and packet collisions. Initially, the cluster will be destroyed after each period to increase the cost of reconstructing the network and affect network stability and, indirectly, power consumption.

### 2.4. WSN Topology Control

Topology control is the most fundamental issue of designing wireless sensor networks studied in [32–38]. The intentions of previous work are to (1) reduce power consumption to prolong network lifetime; (2) decrease electrical influence to increase the effectiveness of MAC and routing protocols; and (3) ensure network connection to guarantee quality of service. How to build an optimal network topology minimizing power consumption is the issue of topology control. There are two types of topology control: power control and state scheduling. The main purpose of power control is to prolong network lifetime by decreasing power consumption by node transmission. However, this focus does not consider network QoS. In the operation of a wireless sensor network, the quantity of power that a sensor requires is the same in the receivable, idle and transmission modes. In the sleep mode, reduced power consumption is a given. Moreover, power consumption increases when the communication areas overlap too much. Nevertheless, state scheduling has obvious benefits in a crowded network topology. In the past, the network connectivity is utilized to evaluate the connection quality [39]. The network is able to build more routes if the connection quality is higher. However, in order to achieve a higher connection quality, the high power consumption is necessary. Hence, in recent years, the network reachability is generally adopted to evaluate the connection quality. The reachability is the percentage of either indirect or direct delivery between all nodes. Each node is able to communicate with distant nodes by multi hops with low power consumption. Hence, from the point of view of energy saving, the reachability is more appropriate to evaluate the connection quality than connectivity.

The power control schemes are classified as power control combined with routing protocol (PCCRP) and power control based on node degree (PCND). COMPOW, which was proposed by Narayanaswamy [40], is a PCND approach. In COMPOW, it is assumed that all nodes are identical in transmission and energy resources. When a node intends to transmit data to a distant node, all nodes will be activated with increased power. This approach is suitable for a WSN with uniform node deployment. Kawadia [41] proposed a power control scheme known as CLUSTERPOW in which each node transmits data in a minimum power layer to the closest neighboring node. To ensure the connection between two neighboring nodes *n_A_* and *n_B_*, the transmission range is determined by the distance between *n_A_* and *n_B_*. Although the necessary power for transmission range is efficiently managed, required computation is still energy-consuming. Ramanathan proposed LMA and LMN algorithms [42] to ensure the node degree within the upper and lower bounds by controlling transmission power. However, the proposal cannot guarantee a fully reachable network.

## 3. A flexible Approach to a Reachable Network Topology with a Minimum Connection Degree

### 3.1. Network Model Design

**Figure 1.**
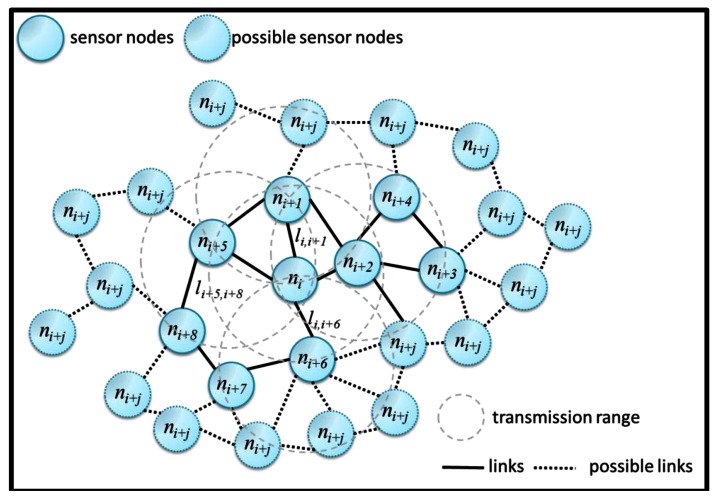
A graph of wireless sensor network.

Network model is essential for this work. A graph-based network model is used to describe a certain network topology. Consider *Ñ* nodes in a network, to save node energy and to prolong network life, not all nodes works concurrently. Sleep schedule algorithm had been applied on sensor nodes to arrange nodes working in turns. Thus, sensor nodes are regarded as two states named as AwakeState and SleepState. The nodes in AwakeState are active to sense and deliver out environmental data. The nodes in SleepState are suspended In the network model, a network, denoted as *Net*, is composed of a set of active node *N* and a link set *L*, *i.e.*, *Net*=*{N,L}* in which *N*=*{n_1_, n_2_, n_3_,…, n_|N|_}* and *L*=*{l_i,j_}* where 1 ≤ *i*,*j* ≤ |N|. The link, *l_i,j_*, stands for the connection between node *n_i_* and *n_j_*. When node *n_j_* is not the neighbor of node *n_i_*, there is no link to deliver data each other directly. They need a set of reply nodes, *{n_k_}*, to set up a data path. Each node *n_i_* has a maximum transmission power *P_tx_* in watts to realize a corresponding maximum transmission distance, *Max_tx*, in meters. The nodes *n_i_* and *n_j_* deliver data directly with each other when the distance of nodes *n_i_* and *n_j_* is less than the Max_tx. If the distance between nodes *n_i_* and *n_j_* is greater than the Max_tx, nodes *n_i_* and *n_j_* have to indirectly communicate each other. The needed transmission power of each node is transmission_range/Max_tx in direct communication. A graph of a static WSN is shown in Figure 1.

Each node connects to other nodes within its transmission range. In real applications, the links between two nodes cold be enabled or disabled for the reasons of energy-saving and link quality. For instance, not all nodes are in work at the same time. Due to the demand of energy-saving, sleeping schedules were applied to schedule nodes either on-duty or off-duty. Consequently, in this work, only enabled nodes are considered to construct a full reachable network.

**Figure 2.**
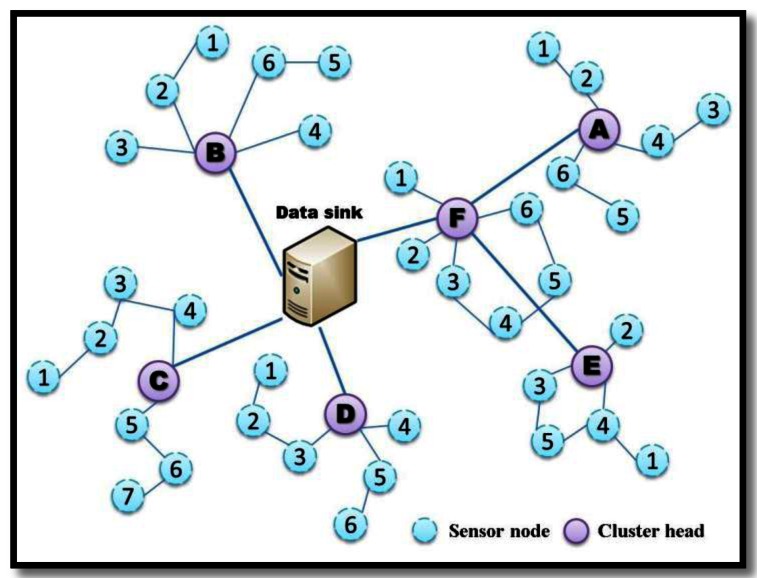
Three-tier network model.

Besides, the number of connected neighboring nodes could be increased as Max-tx increasing. The number of connected neighboring nodes defines the node degree, which is varied with the transmission range of nodes. For an example, the node *n_i_*_+5_, *n_i_*_+1_, *n_i_*_+2_ and *n_i_*_+6_ are the neighbors of *n_i_* and the degree of *n_i_* is equal to 4. In that case, for the node *n_i_*_+7_, the packets from node *n_i_*_+7_ should be forwarded by *n_i_*_+6_. Consequently, we will develop the proposed scheme based on a three-tier network model. The operation of this hierarchical network is shown in Figure 2. The three-tier network model is composed of data sink, cluster head (router), and sensor node (node). The data sink in the first layer stores, processes and maintains data from sensor nodes. The routers in the second layer are the cluster heads to collect data from sensor nodes and forward data to data sinks. The third layer of sensor nodes is in charge of sensing environmental information and delivering data through routers as well as toward data sink.

A component is a node set, in which there is at least a path between two arbitrary nodes. In a component, all nodes can deliver data each other. In a network with only a component, the network topology is full reachability. If the network topology is formed by many components, the network reachability is decreased. Hence, the transmission range is the key point for component number in a network. And the network topologies also are produced through the transmission range. To evaluate the connection quality of a network, three estimated values are adopted, including connectivity, reachability and percentage of k-degree node. Connectivity is defined in relative to the directness of the route between nodes in a network topology. For example, for a network with |*N*| nodes, *N*=*{n*_1_, *n*_2_, *n*_3_….*n_\N\_}*, where the degree of node *(n_j_)* is *deg(n_i_)*, the connectivity is given as:

(1) $$\text{Connectivity}\left(\text{Net}\right)=\sum_{i=1}^{\left|N\right|}\frac{\text{deg}(n_{i})}{2\left(\begin{array}{c} \left|N\right|\\ 2 \end{array}\right)}$$

Accordingly, a network has full connectivity when the network's *Connectivity (Net)* equals to 1. The sufficient and necessary condition of full network connectivity are given as:

(2) $$\sum_{i=1}^{\left|N\right|}\text{deg}(n_{i})=|N{|}^{2}-|N|$$

Full connectivity indicates that the transmission range of network is greater than or equal to the longest distance between any pair of nodes in the network and the degree of each node equals to |*N*|−*1*. The nodes in this network topology can directly exchange data. However, the sufficient transmission power is higher. In addition, this network topology will cause serious channel interference, which decreases the network QoS because the transmission range is too long.

Reachability expresses the indirectness of the route between node pairs in a network topology. For example, in a network with |*N*| nodes where |*N*| nodes are formed of |*G*| partitions *G*=*{g*_1_, *g*_2_, *g*_3_,….,*g*_|_*_G_*_|_*} a*nd *g_j_*=*{ n*_1_, *n*_2_, *n*_3_….*n*_|_*_gj_*_|_*}*, reachability is given as:

(3) $$\text{Reachability}\left(\text{Net}\right)=\sum_{j=1}^{\left|C\right|}\frac{\left(\begin{array}{c} \left|C_{j}\right|\\ 2 \end{array}\right)}{\left(\begin{array}{c} \left|N\right|\\ 2 \end{array}\right)}$$

The requirement of full reachability in network topology is *Reachability* (*Net*) = 1. However, a network only possesses full reachability if a network has only one partition. The proof is as follows.

If the network is fully reachable, we have:

Reachability(Net)=1

That is:

(4) $$\sum_{j=1}^{\left|C\right|}\left(\begin{array}{c} \left|C_{j}\right|\\ 2 \end{array}\right)=\left(\begin{array}{c} \left|N\right|\\ 2 \end{array}\right)$$

Expanding both sides of the equation yields:

(5) $$\sum_{j}^{\left|C\right|}|C_{j}{|}^{2}-\sum_{j}^{\left|C\right|}|C_{j}|={\left|N\right|}^{2}-|N|$$

Because $\left|N\right|=\sum_{j}^{\left|C\right|}|C_{j}|$, we have:

(6) $$\sum_{j}^{\left|C\right|}|C_{j}{|}^{2}-\sum_{j}^{\left|C\right|}\left|C_{j}\right|={\left(\sum_{j}^{\left|C\right|}|C_{j}|\right)}^{2}-\sum_{j}^{\left|C\right|}|C_{j}|$$

Then, the sufficient and necessary condition of full network reachability is:

(7) $$\sum_{j}^{\left|C\right|}|C_{j}{|}^{2}={\left(\sum_{j}^{\left|C\right|}|C_{j}|\right)}^{2}$$

There is at least an indirect or direct route between nodes. In this network topology, the degree of each node is at least greater than or equal to 1. Less power is consumed because of indirect delivery. Channel interference is decreased because the degree of the node is less than that of a node in a network with full connectivity. Therefore, the Qos of network is enhanced.

The percentage of k-degree nodes ( $P_{c}^{k}$) is defined as the proportion of nodes with the k degrees in a network topology. For example, in a network in which *Net*=*{N, L}* with |*N*| nodes and |*L*| links, the node set $N_{k}=\{n_{i}|\text{deg}\left(n_{i}\right)\geq \hat{\text{deg}}(\text{Net})\}$ denotes that the node degree is equal to or greater than the expected node degree $\hat{\text{deg}}(\text{Net})$. Thus, the percentage of k-degree nodes in a network is given as:

(8) $$\text{Pro}\left(\hat{\text{deg}}\left(\text{Net}\right)=k\right)=\frac{\left|N_{k}\right|}{N}$$

The full percentage of k-degree nodes indicates that all nodes in a network satisfy the expected node degree $\hat{\text{deg}}(\text{Net})$. This network will possess full connectivity if $P_{c}^{k}\left(N_{k}\right)=100\%$ and *k*=|*N*|−*1*. Therefore, high percentage of k-degree nodes indicates that a relay node exists in any pair of nodes. The value estimated by Equation (8) will be adopted to verify the reliability of the adjustment model of transmission range.

The transmission range is adjusted by node degree. This adjusted transmission range creates a network topology. In identical node density, the needed transmission range is longer and needs more power for higher node degree. And the number of connected nodes is higher. By contrast, for less node degree, the needed transmission power and the number of connected nodes are less due to shorter transmission range. The network topology is changed by adjusting transmission ranger with required node degree. In different densities, the needed transmission range is diverse in the same node degree. Thus, the node density influences the transmission range in the same node distribution. For example, the distance between nodes in low node density is longer than that between nodes in high node density. As a result, the node degree and node density are significant factors for the determination of the transmission range. For this reason, an important issue is how to determine a proper node degree by adjusting the transmission range for network optimization with different node densities. The network lifetime and operation efficiency are affected by network topology. Therefore, in this study, node degree and node density are considered in designing an adjustment model of the transmission range to establish an optimal, energy-efficient network with full reachability.

### 3.2. Scenario Description

To ensure the data transmission from the sensor nodes to the data sink, full reachability is required for each cluster in the third and second layers. For the sensor node SN_A3, the distance is farther between SN_E3 and the data sink, and high transmission power is required if data are directly delivered from SN_E3 to the data sink. Certain sensors become unable to deliver data to a data sink because of limited transmission power. In another scenario, SN_A3 transmits data to SN_A4, and SN_A4 forwards data to cluster head CH_A. CH_F acts as a relay node between CH_A and the data sink. Therefore, the power consumption from SN_A3 to the data sink is divided between SN_A4, CH_A and CH_F.

In a hierarchical network, multi-hop delivery is an effective approach to equalize the power consumption of nodes in a long transmission. A sufficient condition of fully reachable networks is to ensure that the degree of each node is greater than one. However, the impact of transmission power depends on node degree. Therefore, to save node energy for a node, a proper node degree is required, and the transmission range must be adjusted by node density. According to the above description, a transmission power optimization with a minimum node degree is proposed to construct a fully reachable, energy-efficient network. The proposed approach will be illustrated in the following subsection.

### 3.3. A Full-Reachable WSN Based on an Adaptive Transmission Range with Minimum Node Degree

**Figure 3.**
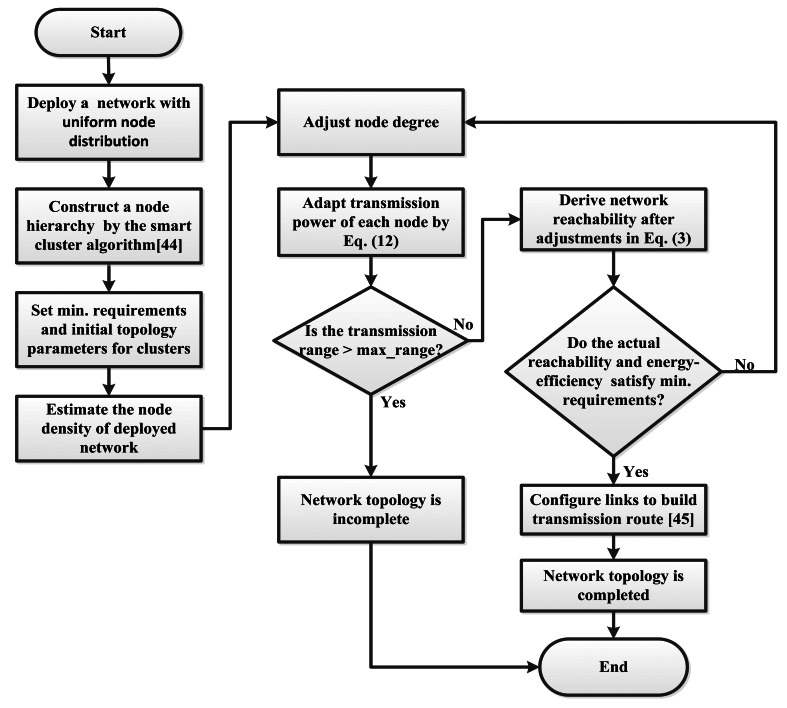
The flowchart of an adaptive optimization transmission with a minimum node degree.

An adaptive approach to support minimum node degree and energy efficiency of WSN is proposed. A flowchart of the proposed approach is shown in Figure 3. The details of the proposed approach will be presented in this subsection. This study mainly emphasizes topology control for power control based on a hierarchical network. Therefore, the optimal transmission range will be obtained according to node degree and node density through the proposed approach. The architecture of the proposed framework is initially composed of the network environment, construction of the hierarchical network, control of the topology network and construction of the transmission route. First, the environmental parameters are set to imitate a real application. Next, the cluster technique [43] is applied to cluster sensor nodes of the target network. The hierarchical network is composed of node clusters to manage the transmission range in a cluster. The transmission range is obtained through the proposed adjustment model of the transmission range to connect adjacent nodes and form a network topology. Thus the proposed approach will contribute an optimal network topology as indicated in Figure 3.

In cooperation with the authors” previous work of the smart cluster algorithm [44] and the routing algorithm [45], a high energy efficiency WSN is obtained through the steps as follows.

Step 1 Initialization of the network environment
  To imitate a real application, the required parameters, including those of the network environment and node specification, are carefully determined. The deployment space is delimited. The maximum transmission powers, power consumption per watt and default node degree for each node are established.
Step 2 Construction of the network architecture
  A smart cluster technique is applied to cluster the network based on the evolutionary computation technique of this framework. To develop the network architecture, information on position and clusters is not known in advance. For the hierarchical network in this study, there are three layers: data sink, routers, and sensor nodes. The data sink is in the first layer. All of the cluster heads in the second layer are included to form a cluster and function as a router. The third layer is composed of groups from clustered sensor nodes. The sensor nodes in a cluster deliver data to the data sink through the corresponding cluster head. The cluster heads transmit or forward data to the data sink.
Step 3 Control of the network topology
  A hierarchical network with nodes organized in clusters is constructed in step 2. In this procedure, an adjustment model of the transmission range is produced to determine an optimal transmission range through the node density, expected node degree, and expected probability of k-degree nodes. The optimal network with energy efficiency and full reachability will be constructed through the derived transmission range.

In the deployment of a wireless sensor network, transmission power should depend on the deployed node density and expected node degree. In other words, transmission power should be adapted according to node sparseness. Thus, Bettstetter [46] proposed a probability of finding an expected node degree (*n*_0_) in two dimensions with uniform distribution and a known node density and transmission radius. The probability is:

(9) $$P_{0}\left(d=n_{0}\right)=\frac{{\left(\rho \pi r_{0}^{2}\right)}^{n_{0}}}{n_{0}!}•e^{-\rho \pi r_{0}^{2}}$$

where *P*_0_*(d* = *n*_0_*)* is the probability of node with *n*_0_ degree

If n_0_ = 0:

(10) $$P_{0}\left(d=0\right)=e^{-\rho \pi r_{0}^{2}}$$

From Equation (10), we have the following equation:

(11) $$\rho \pi r_{0}^{2}-2n_{0}\ln r_{0}-C=0,$$

where *C* = *n*_0_*ln ρ* + *n*_0_*ln π* + *n*_0_*ln n*_0_! − *ln P*_0_

Clearly, we have the transmission range (*r*_0_) as a function of node density, expected node degree and expected probability of k-degree nodes as follows:

(12) $$r_{0}=f\left(\rho ,n_{0},P_{0}\right)$$

The adjustment model as depicted in Equation (12) will be used to determine the optimal transmission range which guarantees the minimum node degree. In implementation, we develop a numerical method to approximate the solution of Equation (12).

For the target network in this procedure, the node density, expected node degree and expected probability of k-degree nodes are required to derive a transmission range through the adjustment mode of the transmission range. Next, the network topology will be established by this transmission range, which is less than or equal to the maximum transmission range. Afterward, connectivity and reachability will be calculated by Equations (1) and (3), respectively. The optimal network is established if the energy savings of a node and the reachability of the network have satisfied minimum requirements. This transmission range is considered the optimal transmission range. Otherwise, the expected node degree will be adjusted to obtain a transmission range capable of satisfying the conditions of an optimal network.

Step 4: Construction of the transmission route
  An intelligent routing algorithm is employed to discover and construct the transmission range based on the swarm intelligence technique after a full-reachability network topology is established. Each node will select one of degrees of node as delivery path. All paths create a delivery route form source node to data sink in a network. A full-reachability is requirement that ensure existent route between source node and data sink. Therefore, in this approach, the transmission range with required node degree is constructed a full-reachability network topology. And based on this network topology, an intelligent routing algorithm is adopted to discover path between nodes to create at least one delivery route from sensor nodes to data sink. Then, other degrees will be backup delivery path. Consequently, this network not only ensures network reachability but also reduces power consumption and channel interference.

**Algorithm 1.** The pseudo code of the adjustment model of the transmission range.
Optimization transmission (density, k-degree probability, initial node degree, maximum transmission range, requirement of energy savings (min_ES), and requirement of reachability (min_R))Start Step 1:  To derive the optimal transmission range *r*_0_ from Equation (12)  If *r*_0_ ≥ maximum transmission range then the network topology is incomplete  Else   r0<maximum transmission range then   Evaluate the network reachability from Equation (3)   energy-savings = pow(transmission range, 2)/pow(maximum range, 2)  End Step 2:  If reachability ≥ min_R and energy-saving ≥ min_ES then the optimal network topology is completed.  Else  degree++;  go to step 1.   EndEnd


## 4. Simulation Results

Simulations were performed to examine the relationship between node degree and transmission range in different node densities. Estimates are adopted to evaluate the power consumption of the nodes in a network and connection quality. The relationship between the performance indices will be clarified by a numerical simulation. Furthermore, the proposed approach enables the proper transmission range to be obtained through the node degree to establish an optimally energy-efficient network with full reachability. The reliability and feasibility of the approach are verified by simulation results.

There are two test environments: low node density with 0.0005 nodes/m^2^ and 0.001 nodes/m^2^ and high node density with 0.005 nodes/m^2^ and 0.01 nodes/m^2^. The other environmental parameters are as follows:

1. The test environment is a 300 m × 300 m region.
2. The generated distribution of nodes is uniform with 0.0005 nodes/m^2^, 0.001 nodes/m^2^, 0.005 nodes/m^2^ and 0.01 nodes/m^2^ on a two-dimensional plane.
3. The expected probability of nodes with k degrees is 100%.
4. The maximum transmission range per watt is 300 m.
5. The initial node degree is 0.

The performance of performance with the different densities is described in four tables, each of which shows the required transmission range and corresponding k-degree probability, energy savings, connectivity and reachability with node degrees. The network performance with low node density is shown in Tables 1 and 2. In Table 1, for a network with full reachability with a node density of 0.0005 nodes/m^2^, the transmission range requires 121.5 m, and the degree of most nodes is less than 4. When the node density is increased from 0.0005 nodes/m^2^ to 0.001 nodes/m^2^, the required transmission range is 64.5 m, and the proportion of k-degree nodes approaches 100%, as shown in Table 3. The energy savings of each node are 83.6% and 95.4%, respectively. For high node density, the network performance is shown in Tables 3 and 4.

A network with high node density only requires a 28.5-m transmission range to establish full reachability. The energy savings are more than 98%. Accordingly, high node density saves more energy than low node density for a network with full reachability. The error of expected probability and actual percentage of k-degree nodes for different node degrees in the node densities are shown in the four tables. The error decreases with increasing node degree. If the node degree is greater than five, the error decreases to zero. This result indicates that the proposed approach is reliable and feasible.

**Figure 4.**
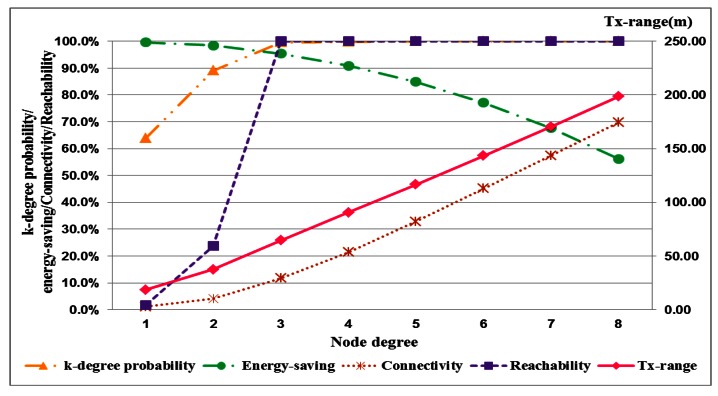
The corresponding relationship of network evaluations with a node density of 0.0005 nodes/m^2^.

**Figure 5.**
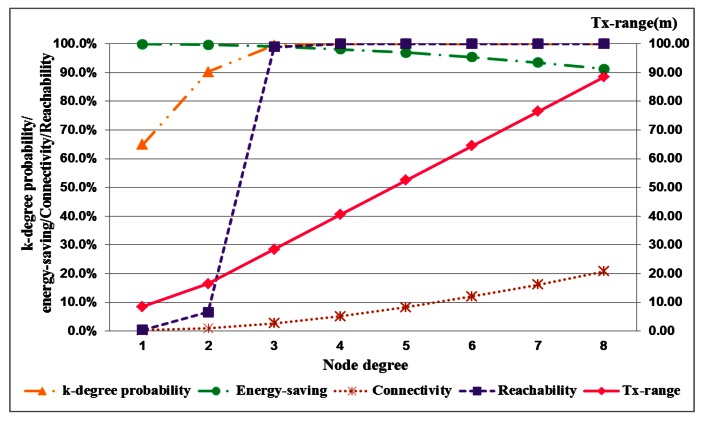
The corresponding relationship of network evaluations with a node density of 0.005 nodes/m^2^.

The performance index curves for low and high node densities are displayed in Figure 4 and 5, respectively. This result implies that the transmission range in low node density is more easily influenced by node degree than that in high node density. A long transmission range is required to connect more adjacent nodes for node. In a uniform node distribution, node density produces different distances between nodes. And the required transmission range is also different because of node density for identical node degree. This result shows that node degree and node density are important factors in the adjustment of the transmission range. And these factors seriously affect energy saving of nodes.

**Figure 6.**
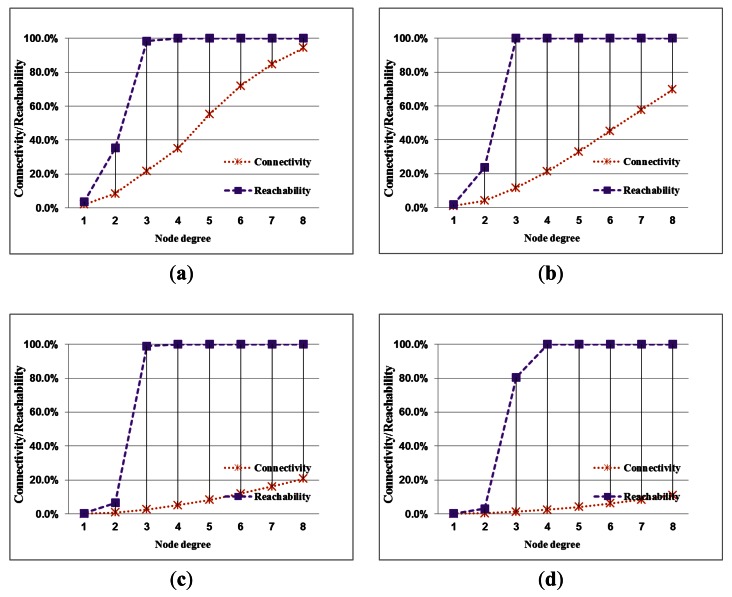
A comparison of the simulation results for connectivity and reachability with different node densities.(**a**) density = 0.0005 nodes/m^2^; (**b**) density = 0.001 nodes/m^2^; (**c**) density = 0.005 nodes/m^2^; (**d**) density = 0.01 nodes/m^2^.

The performance index curves for the connection quality of a network with different node densities are shown in Figure 6. The two curves imply the relationship of reachability and connectivity of network.

**Figure 7.**
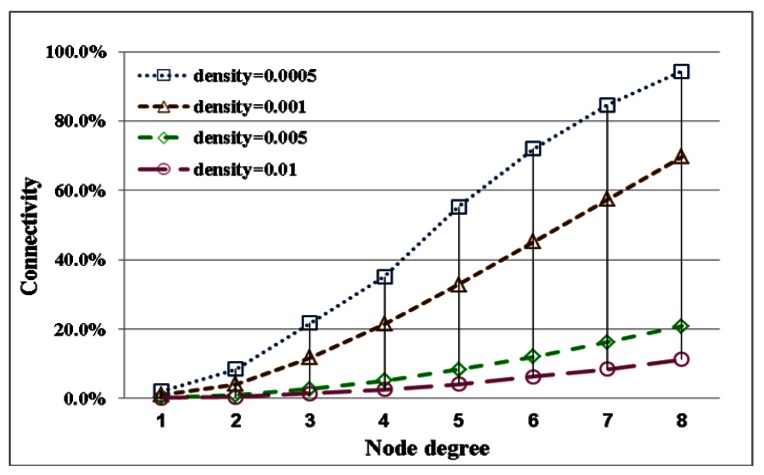
A comparison of the simulation results for connectivity with node degrees ranging from 1 to 8 with different node densities.

The difference between reachability and connectivity becomes apparent by comparing the different node densities. In low nodes density, the two estimates are closer to present that a full-reachability network easily approach to a full-connectivity network. This condition is obvious when the node degree is large. The difference between connectivity and reachability is influenced by node density. Regarding the stability of the evaluation, network reachability maintains stability under different node densities. Therefore, generally, network reachability can serve as a useful indicator of connection quality for a network. The quality of network connectivity with different node densities is shown in Figure 7. This performance index is easily influenced by node density. A network with full connectivity is difficult to construct with high node density. Accordingly, except for special applications, network connectivity does not serve as a useful indicator of connection quality for a network. Instead, reachability is a better index of network topology to reasonably evaluate the performance.

**Figure 8.**
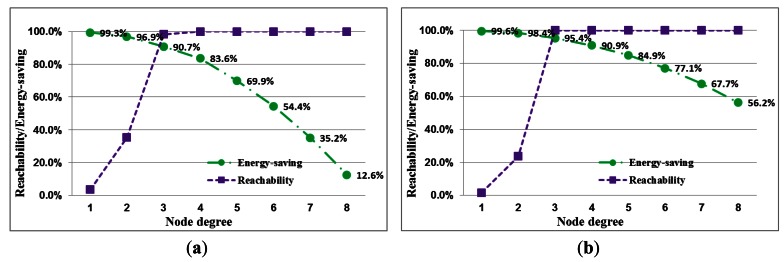

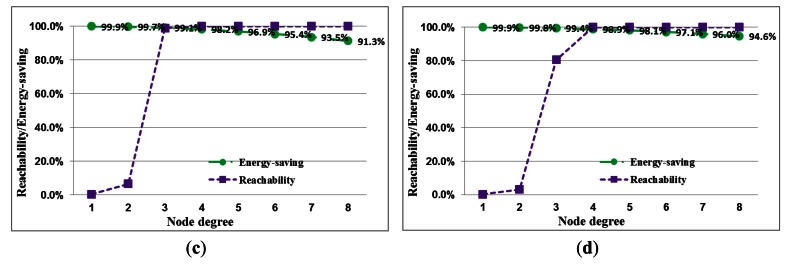
A comparison of the simulation results for energy savings and reachability with different node densities. (**a**) density = 0.0005 nodes/m^2^; (**b**) density = 0.001 nodes/m^2^; (**c**) density = 0.005 nodes/m^2^; (**d**) density = 0.01 nodes/m^2^.

**Figure 9.**
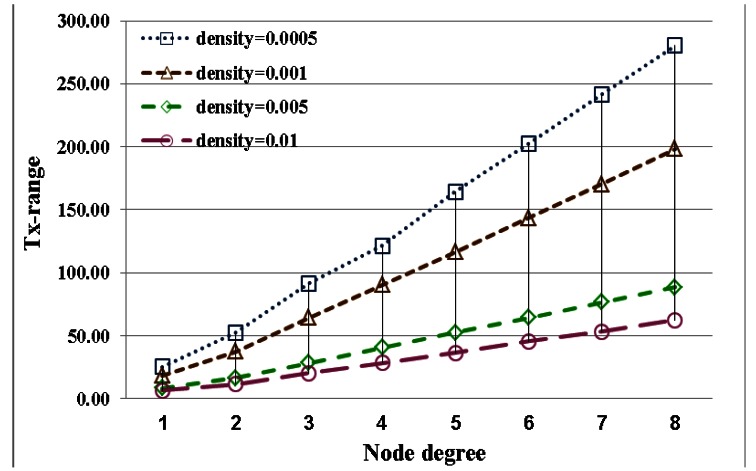
A comparison of simulation results for the transmission range with node degrees ranging from 1 to 8 with different node densities.

**Figure 10.**
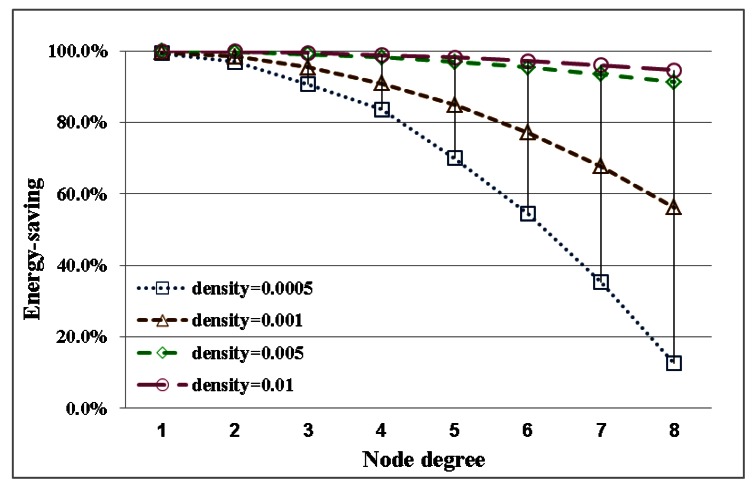
A comparison of simulation results for energy savings with node degrees ranging from 1 to 8 with different node densities.

In this section, the relationship between energy efficiency and transmission range with different densities is discussed. The difference between estimates is shown in Figure 8. Energy savings relate to transmission range. Transmission range is adjusted by node degree and density. Therefore, energy savings are also influenced by node degree and density. For energy efficiency with different node densities, the curves for transmission range and energy savings are shown in Figures 9 and 10, respectively. The variations in energy savings and transmission are more extreme for different node degrees with low node density. In contrast to low node density, the variations in energy savings and the transmission range are slight because the transmission range is harder to influence through node density. In addition, for a network with full reachability, the energy savings of node in high node density are larger than those in low node density.

The node distribution is the critical factor to determine the relation between node degree and network reachability. In nature, the network reachability is increased as the node degree increasing. In this study, the adjustment of the transmission range with a minimum node degree is proposed to guarantee a full reachability network topology. There is no isolated node in a full reachability network topology. In other word, in this network topology, any pair of nodes is able to deliver data each other. In order to achieve this goal, a proper node degree is required. How to obtain a proper node degree to establish a full reachability network topology is a significant issue. In this study, the proposed algorithm first determines the node degree and adjusts the transmission range to make all nodes with this degree. The nodes will construct the links with other nodes based on this derived transmission range. The expected node degree will be adjusted continually until the full reachability network topology is established. All nodes in a network will meet the expected node degree to establish network topology. The network reachability is higher if the expected node degree is increased. When the full reachability network topology is established, the increment of expected node degree is unable to affect the network reachability. The network patterns with uniform distribution are attempted to establish a full reachability network topology in this study. And the needed node degree is about 3 or 4 to establish a full reachability network topology for these patterns. Hence, the network reachability is changed obviously in the node degree less than 3. The change of network reachability is insignificant when the node degree greater than 4.

Naturally, the network reachability is increased as the node degree increases. As varying with the node distributions, there exists a threshold of node degree affecting the network reachability. In the studied cases, most big jumps occur when the expected node degree is changed from 2 to 3. That means the threshold of node degree is between 2 and 3. Upon the node degree exceeding the threshold, the network reachability approaches to full reachability. This finding benefits the parameter configuration in real applications of the proposed scheme. Regarding to the impact of node distribution on the threshold, we will explore in future work.

## 5. Characteristics Analysis with Uniform Node Distribution

In this section, the characteristics analysis with uniform node distribution is presented. In a network with identical node density, the node degree will affect transmission range and energy efficiency. The transmission range must be adjusted when nodes have to alter degree to achieve optimal connection quality. The node with less degree requires lower transmission power. The node of less degree easily leads to an interruption of the delivery route for a network. In order to maintain the transmission route between node pairs, the transmission range of a node should be increased to facilitate connection with adjacent nodes and provide the possibility of route selection. On the contrary, longer transmission range is necessary to add degree of node but needed power is higher. More node degree will provide more possibility of route selection. However, redundant node degree maybe causes the channel interference. Hence, a proper node degree is extremely valuable for network performance. An optimal transmission range is obtained by proposed adjustment model. A full reachability network with minimum node degree will be built by this optimal transmission range. This optimal transmission how to transform to needed power depends on the chip design. This issue has to be considered in real operation. However, in this study, the proposed mathematical model focuses on theoretical analysis. And the analyzed results will benefit the future deployment of real applications.

Next, the case of a network with different node densities will be discussed. In real applications, node density must be determined first. The distance between nodes is different according to node density. The distance between nodes in low node density is longer than that between nodes in high node density. Hence, the needed transmission power in low node density is higher than that between nodes in high node density under the same node degree. In other words, where node degrees are identical, and each node has to meet this requirement, a network with low node density requires a longer transmission range and higher transmission power than does one with high node density. In addition, the distance between nodes will be closer when node density continuously increases. Accordingly, in a network with high node density, a fixed transmission range can simultaneously satisfy different conditions of node degree. Therefore, the transmission range is not easily influenced by node degree in a network with high node density. This fact indicates that a network with high node density is more efficient to improve energy efficiency. By contrast, the node degree easily influences the transmission range of the nodes in a network with low node density.

There are two performance indices to evaluate the connection quality of network. In this paper, connectivity and reachability are defined according to the directness or indirectness of the route between nodes in a network, respectively. If a node wants to deliver data to a distant node, the power consumption required by direct transmission is higher than that required by indirect transmission. Therefore, a network with full connectivity requires more power than does a network with full reachability. According to the simulation results of Section 4, a network with high node density will improve energy consumption of nodes efficiently. Because of the distance between close nodes is shorter, the needed power for delivery is lower. Hence, each node only connects with adjacent nodes through adjusted transmission range with a proper node degree. This proper node degree has to guarantee to establish a full-reachability network. In this way, the connection of node with far node only needs less power due to indirect delivery. In addition, a network with low node density is difficult for establishing full-reachability network topology. Even if a full-reachability network topology is established, the network is not energy efficient because the full-reachability network and full connectivity network in lower node density are more similar than those in higher node density. Therefore, generally, a network topology with full-connectivity is not recommended.

As a result, node density and node degree are the most significant factors affecting transmission range adjustment in the construction of an optimal network with energy efficiency and full reachability. Ant the transmission range is the most important factor influencing network performance. The trend of these estimates reflects the variation of node degree in different node densities. The difference between these estimates is small in a network with low node density but extreme in a network with high node density. In a high-density network, the transmission range is not easy influenced by node degree in the construction of a network with full reachability. However, network reachability is a more suitable indicator of connection quality of a network than is network connectivity. In this study, an approach is proposed for transmission optimization with a minimum node degree for a network topology that is energy-efficient and fully reachable. Reachability is recommended as an indicator of the connection quality of a network.

## 6. Conclusions

Power optimization is a valuable technique for extending the lifetime and improving the connection quality of wireless sensor networks. Optimized transmission power benefits network performance. In addition, an optimal network draws on the adjustment of transmission power. Therefore, how to tradeoff between prolonging network lifetime and maintaining connection quality is a challenge. In this study, an optimization approach for wireless sensor networks based on a hierarchical network is developed with a focus on topology control of the transmission range. Considering network lifetime and connection quality simultaneously, the transmission range with a minimum node degree is optimally adjusted according to node degree and node density to establish an optimal network with energy efficiency and full reachability. This approach facilitates the selection of a transmission range in the deployment of a wireless sensor network. Although the continuous transmission range is hardly available in real applications due to only discrete levels of transmission power commonly affordable in industrial product such as the cc2530 Zigbee chip from Texas Instrument, the proposed algorithm is applicable to determine the transmission power level.

The reliability and feasibility of the proposed approach are shown by the simulation results and performance indices used to evaluate the energy efficiency and connection quality of a network. The relationship between these performance indices is analyzed to generalize characteristics for low and high node densities. These characteristics analyses inform the deployment strategy of a wireless sensor network. In addition, the practicability of network connectivity and network reachability are compared. As a result, network reachability is a better indicator of connection quality than is network connectivity. These results are a helpful contribution to wireless sensor network design. However, in this study, the power model is incomplete because it disregards the data model, environmental impact, and transmission frequency, among other factors, although transmission power is employed to calculate energy efficiency. In the future, we will consider the effect of factors such as data model, environmental impact on transmission to design a power consumption model for nodes with which network energy efficiency can be evaluated. Appropriate transmission strategies will be suggested for different network topologies.

## Figures and Tables

**Table 1.** The numerical evaluation results for a network with a node density of 0.0005 nodes/m^2^.

	**Node density [0.0005]**				

**Node Degree**	**Tx_Range (m)**	**k-Degree Percentage**	**Energy Savings**	**Connectivity**	**Reachability**
1	25.50	62.7%	99.3%	2.1%	3.5%
2	52.50	86.2%	96.9%	8.4%	35.3%
3	91.50	96.0%	90.7%	21.8%	98.3%
4	121.50	97.8%	83.6%	35.1%	100.0%
5	164.50	100.0%	69.9%	55.3%	100.0%
6	202.50	100.0%	54.4%	72.0%	100.0%
7	241.50	100.0%	35.2%	84.7%	100.0%
8	280.50	100.0%	12.6%	94.3%	100.0%

**Table 2.** The numerical evaluation results for a network with a node density of 0.001 nodes/m^2^.

	**Node density [0.001]**				

**Node Degree**	**Tx_Range (m)**	**k-Degree Percentage**	**Energy Savings**	**Connectivity**	**Reachability**
1	18.50	64.0%	99.6%	1.1%	1.8%
2	37.50	89.1%	98.4%	4.1%	23.7%
3	64.50	99.6%	95.4%	11.7%	100.0%
4	90.50	99.8%	90.9%	21.4%	100.0%
5	116.50	100.0%	84.9%	32.9%	100.0%
6	143.50	100.0%	77.1%	45.2%	100.0%
7	170.50	100.0%	67.7%	57.5%	100.0%
8	198.50	100.0%	56.2%	69.7%	100.0%

**Table 3.** The numerical evaluation results for a network with a node density of 0.005 nodes/m^2^.

	**Node Density [0.005]**				

**Node Degree**	**Tx_Range (m)**	**k-Degree Percentage**	**Energy Savings**	**Connectivity**	**Reachability**
1	8.50	64.9%	99.9%	0.2%	0.4%
2	16.50	90.3%	99.7%	0.9%	6.6%
3	28.50	99.5%	99.1%	2.7%	99.0%
4	40.50	100.0%	98.2%	5.1%	100.0%
5	52.50	100.0%	96.9%	8.3%	100.0%
6	64.50	100.0%	95.4%	12.0%	100.0%
7	76.50	100.0%	93.5%	16.2%	100.0%
8	88.50	100.0%	91.3%	20.8%	100.0%

**Table 4.** The numerical evaluation results for a network with a node density of 0.01 nodes/m^2^.

	**Node density [0.01]**				

**Node Degree**	**Tx_Range (m)**	**k-Degree Percentage**	**Energy Savings**	**Connectivity**	**Reachability**
1	6.50	73.8%	99.9%	0.1%	0.3%
2	11.50	90.4%	99.8%	0.4%	3.1%
3	20.50	99.7%	99.4%	1.4%	80.6%
4	28.50	100.0%	98.9%	2.6%	100.0%
5	36.50	100.0%	98.1%	4.1%	100.0%
6	45.50	100.0%	97.1%	6.3%	100.0%
7	53.50	100.0%	96.0%	8.4%	100.0%
8	62.50	100.0%	94.6%	11.2%	100.0%
